# Modulation of Glutathione-S-Transferase by Phytochemicals: To Activate or Inhibit—That Is the Question

**DOI:** 10.3390/ijms26157202

**Published:** 2025-07-25

**Authors:** Irina Anna-Maria Stoian, Adelina Vlad, Marilena Gilca, Dorin Dragos

**Affiliations:** 1 Department of Functional Sciences I/Biochemistry, Faculty of Medicine, Carol Davila University of Medicine and Pharmacy, 050474 Bucharest, Romania; irina.stoian@umfcd.ro; 2 Department of Functional Sciences I/Physiology, Faculty of Medicine, Carol Davila University of Medicine and Pharmacy, 050474 Bucharest, Romania; adelina.vlad@umfcd.ro; 3 Department of Medical Semiology, Faculty of Medicine, Carol Davila University of Medicine and Pharmacy, 020021 Bucharest, Romania; dorin.dragos@umfcd.ro; 4 1st Internal Medicine Clinic, University Emergency Hospital Bucharest, Carol Davila University of Medicine and Pharmacy, 050098 Bucharest, Romania

**Keywords:** glutathione-S-transferase, phytochemicals, medicinal plants, phase II detoxification

## Abstract

Glutathione S-transferases (GSTs) are phase II detoxification enzymes that display several enzymatic activities, including transferase, peroxidase, reductase, and isomerase functions, as well as non-enzymatic roles (e.g., serving as binding proteins). Their complex functionality lies in the biotransformation of xenobiotics (e.g., pesticides, drugs) and certain endogenous compounds, primarily metabolites produced by phase I detoxification enzymes. Several plant-derived compounds have been shown to modulate the activity and expression levels of these enzymes. Phytochemical activators of GSTs are potentially beneficial for detoxification in cases of exposure to various toxic compounds, whereas inhibitors of GSTs could have positive effects as adjuvant treatments for cancers that express high levels of GSTs associated with drug resistance.

## 1. Introduction

Glutathione-S-transferases (GSTs) are multifunctional detoxification proteins [1]. They are especially known as members of the phase II detoxification enzyme family. In this regard, GSTs catalyze the conjugation of glutathione (GSH) to various electrophilic substrates, both endogenous (e.g., by-products of reactions involving reactive oxygen species) and exogenous (e.g., polycyclic aromatic hydrocarbons epoxides, usually derived from cytochrome P450 metabolism) [2,3,4]. Nevertheless, they also play non-enzymatic roles [1].

GSTs are classified into three families based on their cellular location: cytosolic, mitochondrial, and microsomal. The cytosolic GSTs include eight classes that differ in their amino acid sequences and substrate specificities: Alpha (A), Pi (P), Mu (M), Omega (O), Sigma (S), Theta (T), and Zeta (Z). The mitochondrial family consists of a single class (Kappa, K), while the microsomal GSTs belong to the membrane-associated proteins in eicosanoid and glutathione metabolism (MAPEG) family [3,5,6]. Several distinct gene families encode GSTs on different chromosomes: chromosome 6-GST A, chromosome 22-GST M, chromosome 11-GST P, chromosome 14-GST Z, chromosome 4-GST S, chromosome 10-GST O, while the chromosomal location for GST K remains unknown [6].

GSTs are expressed at high levels in various organs and display a wide range of biological actions (Table 1). Although the main common role of GSTs is to catalyze crucial steps in the metabolism of xenobiotics (detoxification or bioactivation), some GSTs are also involved in more specific functions such as eicosanoid synthesis, steroid hormone metabolism, amino acid degradation, or acting as binding proteins [7,8].

GSTs are characterized by significant structural and functional heterogeneity. For instance, the membrane-bound microsomal GSTs are homo-and heterotrimers involved in the endogenous metabolism of leukotrienes and prostaglandins, whereas the cytosolic GSTs are dimers and participate in the regulation of the mitogen-activated protein kinase (MAPK), c-Jun N-terminal kinase 1 (JNK1), and apoptosis signal-regulating kinase 1 (ASK1) pathways) [3].

GST mRNA and protein levels are elevated in response to various stressors (e.g., oxidant agents, xenobiotics), displaying a cytoprotective effect on stressed human cells such as retinal pigment epithelium, renal tubular epithelium, and neuronal cells [9,10,11].

**Table 1:** Types of glutathione-S-transferases and their distribution and functions.

Enzyme	Variants	Distribution	Specific Functions
GST A	GST A1-1	liver, intestine, kidney, adrenal gland, and testis [12]	detoxification of carcinogenic environmental pollutants, and alkylating chemotherapeutic agents; peroxidase activity toward hydroperoxides of fatty acids and phosphatidyl moieties [12]; binding the mitogen-activated protein (MAP) kinases JNK1 [13]
	GST A2-2	liver, intestine, kidney, adrenal gland, and testis [12]	similar functions with GST A1-1, but to a lesser extent [12]
	GST A3-3	steroidogenic tissues (gonades, mammary gland, placenta, adrenals), lung, stomach, trachea [12]	_ Δ^5^ − Δ^4^ isomerization of steroids [12]
	GST A4-4	many tissues [12]	conjugation of 4-hydroxynonenal [12]
GST P	GST P1-1	brain, heart, lung, testis, skin kidney, pancreas, erythrocytes (GST P is the most prevalent non-hepatic isoenzyme [1])	detoxification of endogenous and exogenous toxic compounds by glutathione-dependent conjugation or by acting as a ligandin [8]; binding the mitogen-activated protein (MAP) kinases JNK1, ASK1, and MEKK1 [2,14]; monomeric GST P acting as an inhibitor of C-jun N-terminal kinase [15]; deletion of GST P1 gene-risk factor of acute leukemia [16]
GST M	GST M1-1	liver (present in some, but not all, liver specimens), GSTM1a and M1b subunits in muscle, testis, and brain [17]; parathyroid [18]	conjugation of prostaglandin A2 and prostaglandin J2 with glutathione (GST M1a-1a) [19]; participating in the formation of novel hepoxilin regioisomers [20]; GST M1: metabolism of isothiocianates (most efficient, among GSTM1, GSTP1, GSTA1, and GSTM4) [21]; binding the mitogen-activated protein (MAP) kinases JNK1, ASK1, and MEKK1 [2,14,22]; deletion of GST M1 gene-risk factor of acute leukemia [16]
	GST M2-2	muscle, testis, and brain [17]	participating in the formation of novel hepoxilin regioisomers [20];
	GST M3-3	muscle, testis, and brain [17] testis, epididymis, seminal vesicles, renal tubules.	no specific function evidentiated; common function of all GSTs (conjugation of reduced glutathione to exogenous and endogenous hydrophobic electrophiles) [23]
	GST M4-4	kidney, intestines, muscle, testis, and brain [17]	glutathione-dependent conjugation of leukotriene A4 (to form leukotriene C4), (S),14(S)-epoxy-docosahexaenoic acid (to form maresin conjugate in tissue regeneration 1 or MCTR1, a potent anti-inflammatory lipid mediator) [24]; metabolism of isothiocyanates (less efficient than GST M1) [21]
	GST M5-5	muscle, testis, and brain [17]	no specific function evidentiated; common function of all GSTs (conjugation of reduced glutathione to exogenous and endogenous hydrophobic electrophiles) [23]
GST T	GST T1-1	kidney, liver, erythrocytes [25], parathyroid [18]	biotransformation of several industrial chemicals (e.g., butadiene, methyl chloride, dichloromethane, epoxides) [25]
	GST T2-2	Skin, brain [23]	no specific function evidentiated; common function of all GSTs (conjugation of reduced glutathione to exogenous and endogenous hydrophobic electrophiles) [23]
GST S	GST S1-1	spleen, hematopoietic system (antigen-presenting cells, Th2 lymphocytes, mast cells, and megakaryocytes) [26]	hematopoietic prostaglandin D synthase (HPGDS) (glutathione-dependent key enzyme in the synthesis of the D and J classes of prostanoids) [27]
GST Z	GST Z1	liver, muscles, testis, brain [23]	glutathione-dependent biotransformation of xenobiotic α-haloacids (e.g., oxygenation of dichloroacetic acid to glyoxylic acid); cis-trans isomerization of maleylacetoacetate (product of tyrosine degradation) to fumarylacetoacetate [7]; glutathione peroxidase (GPx) activity with t-butyl and cumene hydroperoxides [23];
GST K	GST K1-1	low tissue specificity [23]	glutathione-dependent conjugation of 1-chloro-2,4-dinitrobenzene [23]
GST O	GST O1-1	expressed in most human tissues [28], highest expression in the liver, skeletal muscle, and heart [23,28]	glutathione-dependent thiol transferase; dehydroascorbate reductase activities; biotransformation of inorganic arsenic, reduction in monomethylarsonic acid, dimethylarsonic acid [23], reduction in α-haloketones to nontoxic acetophenones [29]
	GST O2-2	liver, kidney, skeletal muscle, testis, lower expression in the heart, cervix, ovary and prostate [30]	thiol transferase activity, reductase activity (reduction in monomethylarsonate and dehydroascorbate) [29]
MGST	MGST1	adipose tissue, adrenal gland, liver, monocytes [23]	glutathione-dependent conjugation of halogenated hydrocarbons, reduction in phospholipid hydroperoxides [31]; denitration of glyceryl trinitrate [32]
	MGST2	liver, intestines, dendritic cells [23]	LTC4 synthase activity [33]
	MGST3	low tissue specificity [23]	LTC4 synthase activity and GPx activity [34]
	PGES-1		biotransformation of arachidonic acid metabolite prostaglandin endoperoxide H2, produced by cyclooxygenase, to prostaglandin E2 [35].
	FLAP	neutrophils [36]	5-lipoxygenase-activating protein, no intrinsic catalytic activity, binding arachidonic acid [36]

Legend: GPx—glutathione peroxidase; MGST—microsomal glutathione-S-transferase; PGES—1-prostaglandin E2 synthase 1.

Several diseases have been linked to GSTs, including neoplastic and inflammatory disorders associated with cytosolic GSTs, as well as respiratory, cardiovascular, and neurovascular disorders related to MAPEG proteins [5]. Consequently, the induction of GSTs plays an important role in cancer chemoprevention [37], and GSTs have been targeted in the treatment of both proliferative [38] and inflammatory disorders [35].

Medicinal and edible plants produce a rich variety of phytochemicals, representing a valuable reservoir for the development of nutraceuticals and functional foods. These naturally occurring compounds are well known for their antioxidative, anti-inflammatory, and disease-preventive activities, and notably, several phytochemicals have demonstrated the ability to influence the activity and expression of human GSTs [5,39].

The purpose of this article is to review the available information on phytochemicals with the potential to modulate glutathione-S-transferase activity and to identify which interventional strategy—activation or inhibition of GSTs?—may be the most appropriate therapeutic choice in different pathological conditions.

## 2. Materials and Methods

Phytochemicals with glutathione-S-transferase stimulatory and inhibitory activity were identified through searches in three international databases: PubMed, Google Scholar, and ScienceDirect. A systematic literature review was conducted using the following search string: glutathione-S-transferase AND (phytochemical OR phytocompound OR medicinal plant OR herb) AND (activate OR inhibit). Additionally, a manual search was performed to include as many relevant phytocompounds as possible in our study. No time limit was imposed on the publication dates of the papers covered in the review. The literature search was initially performed in February–March 2023, then updated in September–October 2024, and most recently in April–May 2025, resulting in 141 articles included in this review. The data presented reflect the information available at the time the paper was prepared.

Only individual phytocompounds (used alone or in combination) are considered in this review. Plant extracts were excluded as it is not possible to ascertain with sufficient confidence which specific phytochemical(s) are responsible for the investigated effects.

One paper was excluded due to a discrepancy between the conclusions (which asserted a return of GST levels to normal) and the raw data (which showed a decrease in GST levels after administration of the active phytocompound) [40].

## 3. Glutathione-S-Transferase Activation by Phytochemicals

GSTs metabolize not only chemotherapeutic agents and carcinogens, but also metabolites derived from oxidative stress [17]. Given their potential therapeutic role in some of the most prevalent diseases with high morbidity and mortality, inducers of GSTs have been sought-and identified-in the vegetal realm. Phytochemicals that have demonstrated GST-activating potential are summarized in Table 2. The majority of these phytochemicals (e.g., anthocyanins, isothiocyanates, gallic acid, 6-shogaol, curcumin, resveratrol) are well known for their cancer-preventive effects [41,42,43,44] and for their ability to increase the expression or activity of GST. This, in turn, enhances the liver’s capacity to neutralize chemical carcinogens [45,46,47], protects it from the damaging effects of various pro-oxidant xenobiotics [44,48], or enables it to compete with and outcompete-potential toxins (e.g., acrylamide) for biotransformation by GST [37].One of the mechanisms involved in antioxidant chemoprevention is the activation of the antioxidant response element (ARE), which consequently induces the expression of enzymes involved in glutathione metabolism, including not only GSTs but also GR and GPx [44,49].

**Table 2:** Phytochemicals with stimulatory activity on glutathione-S-transferase.

Phytochemicals (Source/Chemical Subclass)	Mechanisms of Action	Type of Study	References
**Alkaloids**			
indole-3-acetonitrile (indole alkaloid)	increased GST activity	(in vivo) liver and small intestine in female ICR/Ha mice	[50]
boldine (*Peumus boldus* Mol.) (aporphine alkaloids, a sub-class of quinoline alkaloids)	stimulated GST activity	(in vitro) mouse hepatoma Hepa-1 cell line	[51]
clivorine (*Ligularia hodgsonii* Hook, *Ligularia dentata* Hara) (pyrrolizidine alkaloid)	4 times increase in GST activity by about 400%	(in vitro) L-02 cells (derived from adult human normal liver) treated for 48 h with 50 mM clivorine	[52]
berberrubine (proto-alkaloid of berberine, a benzyl-isoquinoline alkaloid)	GSTM2 promoter activation leading to increased GSTM2 mRNA and protein expression	(in vitro) bladder cancer cell lines, 5637 and BFTC 905	[53]
**Coumarins**			
coumarin (benzopyrone)	increased class-pi GST P1 subunit	(in vivo) treatment for 2 weeks before exposure, continued during exposure, prevented aflatoxin B1-induced hepatocarcinogenesis in rat;	[54]
	increased GST activity	(in vivo) liver and small intestine in female ICR/Ha mice	[50]
fraxetin [*Fraxinus chinensis* subsp. *rhynchophylla* (Hance) A.E.Murray]	concentration-dependently increased GST A, but not GST M or GST P levels	(in vitro) transiently transfected H4IIE cells (rat hepatoma); Fraxetin (10–100 mM)	[55]
**Terpenes/terpenoids**			
limonene (essential oil extracted from *Citrus* fruits pericarp) (cyclic monoterpene)	increased GST activity	(in vivo) larvae and adult *Drosophila melanogaster* flies fed with 5 mL of 0.25%, *v*/*v* solution (in distilled water) and urethane (20mM) (as a genotoxic agent)	[56]
p-mentha-2,8-dien-1-ol (seed oil from *Apium graveolens* L.) (monocyclic monoterpenoid)	increased GST activity	(in vivo) female A/J mice, liver and small intestinal mucosa; 20 mg/dose every two days for a total of 3 doses	[57]
D-limonene (abundant in *Citrus* plants like lemon, orange, and grape) (monoterpene)	increased GST activity	(in vivo) male Albino Wistar rats with streptozotocin (STZ) (40 mg/kg i.p.) -induced diabetes in; D-limonene 100 mg/kg BW for 45 days	[58]
geraniol (many herbs, e.g., lavender, citronella, lemongrass) (monoterpenoid alcohol)	increased activity of GST (probably by upregulated expression)	(in vivo) animal model of isoproterenol-induced myocardial infarction	[59]
β-caryophyllene (bicyclic sesquiterpene)	Reverses isoproterenol-induced GST inhibition	(in vivo) rats with isoproterenol (100 mg/kg body weight) -induced myocardial infarction; β-Caryophyllene 20 mg/kg body weight daily pre-and co-treatment orally, for 3 weeks	[60]
β-caryophyllene [1], β-caryophyllene oxide [2], α-humulene [3], α-humulene epoxide I [4], eugenol [5] [*Syzygium aromaticum* (L.) Merr. & L.M.Perry] (sesquiterpene)	induction of GST in the mouse liver and small intestine	(in vivo) female A/J mice were given by gavage 20 mg per dose of the test compounds once every 2 days for a total of three doses	[61]
cycloartenol (abundant in fruits, vegetables, medicinal plants) (triterpenoid sterol)	partial recovery of GST activity depressed after exposure	(in vivo) GST activity decreased in the skin of mice exposed to benzoyl peroxide treatment (20 mg/animal/0.2 mL acetone) and UVB radiation (0.420 J/m(2)/s)	[62]
kahweol/cafestol (*Coffea arabica* L. coffee) (diterpenoid alcohol)	hepatic GST activity/expression increased (overall GST, GST A, GST M, GST P, GST T)	(in vivo) male Fisher F344 rat fed kahweol/cafestol at 0.122%, for 10 days	[63,64]
lupeol (found in many fruits and medicinal plants) (pentacyclic triterpene)	increased activity of GST in murine skin	(in vivo) prophylactic treatment of mice with lupeol (0.75 and 1.5 mg per animal) 1 h before benzoyl peroxide (a cutaneous tumor promoter)	[65]
lupeol (3’-hydroxylup-20(29)-ene) [*Cyanthillium cinereum* (L.) H.Rob., little ironweed] (pentacyclic triterpenoid)	prevention of the GST activity reduction selenite-induced	(in vivo) 10, 25, 50 mg/g body weight given orally from the 8th day up to the 21st day to Sprague Dawley rat pups to prevent selenite-induced cataract	[66]
deacetyl nomilin (*Citrus* × *aurantium* L., sour orange seed powder) (citrus limonoid/triterpenoid)	induction of GST	(in vivo) Female A/J mice, small intestine and liver; 20 mg by oral gavage once every two days, four administrations	[67]
isoobacunonic acid (*Citrus* × *aurantium* L., sour orange seed powder) (citrus limonoid/triterpenoid)	induction of GST	(in vivo) Female A/J mice, small intestine and liver; 20 mg by oral gavage once every two days, four administrations	[67]
nomilin (*Citrus* × *aurantium* L., sour orange seed powder) (citrus limonoid/triterpenoid)	induction of GST	(in vivo) Female A/J mice, small intestine and liver; 20 mg by oral gavage once every two days, four administrations	[67]
**Flavonoids**			
flavone (found in various amounts in most plants) (flavone)	GST induction (GST A > GST M > GST P) potentially chemopreventive in the stomach, small intestine, liver, and to a lesser extent in the esophagus	(in vivo) male Wistar rats	[68]
baicalein (root of *Scutellaria baicalensis* Georgi., Baikal skullcap) (flavone)	GSTM2 promoter activation leading to increased GSTM2 mRNA and protein expression	(in vitro) bladder cancer cell lines, 5637 and BFTC 905	[53]
wogonin (root of *Scutellaria baicalensis* Georgi., Baikal skullcap) (flavone)	GSTM2 promoter activation leading to increased GSTM2 mRNA and protein expression	(in vitro) bladder cancer cell lines, 5637 and BFTC 905	[53]
puerarin [from the roots of *Pueraria montana* var. *lobata* (Willd.) Maesen & S.M.Almeida ex Sanjappa & Predeep, kudzu vine] (isoflavone)	increased GST level depressed by cisplatin	(in vivo) model of rat (female Sprague-Dawley) cisplatin toxicity; 50 mg/kg puerarin	[69]
quercetin [found in many plants, such as grapes, onions, green tea, apples, berries, etc.] (flavonol)		(in vivo) larvae and adult *Drosophila melanogaster* flies fed with 5 mL 0.25%, *w*/*v* solution (in distilled water) and urethane (20mM) (as a genotoxic agent)	[56]
naringenin (common in citrus fruits) (flavanone)	increased GST activity increased transcription of the *GST* gene	(in vitro) MIN6 (mouse insulinoma cell line); (in vivo) animal model of streptozotocin-induced diabetes (in vivo) Sprague-Dawley rats treated with 50 and 100 mg/kg body weight of naringenin for 7 days	[70], [71]
	increase in GSTa3 cDNA; best protective effect with the 50 mg/kg body weight dose		
β-naphthoflavone (synthetic flavonoid) (flavone)	increased GST activity	(in vivo) liver and small intestine in female ICR/Ha mice	[50]
morin (*Maclura pomifera* (Raf.) C.K.Schneid., Osage orange) (flavonol)	enhanced expression of GST (effective concentration 60 µM)	(in vitro) L6 myotubes treated cells.	[72]
chalcone (many sources) (chalcone)	GSTM2 promoter activation leading to increased GSTM2 mRNA and protein expression	(in vitro) bladder cancer cell lines, 5637 and BFTC 905	[53]
anthocyanidins (cyanidin, delphinidin, malvidin), anthocyanins (cyanidin-3-O-glucoside = kuromanin) [red, purple, blue, or black fruits (grapes, blueberries, etc.) and vegetables (purple cabbage, red onion, radishes, etc.)](flavonoid)	GST induction by activation of ARE	(in vitro) rat liver Clone 9 cells	[44]
**Other phenolics**			
gallic acid (found in various amounts in most plants) (phenolic acid)	GST induction potentially useful for preventing hepatotoxicity; increased activity levels of GST	(in vivo) cyclophosphamide-induced hepatotoxicity in male Wistar rats treated with 60 and 120 mg/kg body weight for 14 days, orally;	[48]
		(in vivo) larvae and adult Drosophila melanogaster flies fed with 5 mL 0.5%, w/v solution (in distilled water) and urethane (20mM) (as a genotoxic agent)	[56]
6-shogaol [*Zingiber officinale* Roscoe, ginger] (phenolic compound)	GST induction potentially useful for preventing colorectal cancer	(in vivo) adult male mice with colorectal adenoma induced by azoxymethane and dextran sulfate sodium, treated with 20 mg/kg BW for 21 days	[73]
**Isothiocyanates**			
allyl isothiocyanate (*Brassica oleracea* var. *gemmifera* DC., Brussels sprouts)	GST induction in liver and small intestine	(in vivo) male Fisher rats	[74]
benzyl isothiocyanate (common in Brassicaceae vegetables, such as broccoli, cabbage or watercress)	increased GST activity	(in vivo) liver and small intestine in female ICR/Ha mice	[50]
benzyl isothiocyanate (common in Brassicaceae vegetables, such as broccoli, cabbage or watercress)	GST P rapidly synthesized in hepatocytes, and rapidly excreted into bile (evidentiated by immunostaining with GST-P antibody)	(in vivo) Male Sprague–Dawley rats fed a basal diet containing BITC (0.5%) ad libitum.	[75]
4-methylsulfanyl-3-butenyl isothiocyanate [derived from glucoraphanin (present in daikon (*Raphanus sativus* var. *longipinnatus*, *L.H.Bailey*) sprouts) by the action of myrosinase isolated from *Sinapis alba* L., white mustard]	increased GST activity	(in vitro) liver slices from Male Wistar albino rats incubated for 24 h with glucosinolate (0–10 μM) + myrosinase (0.018 U)	[45]
phenethyl isothiocyanate (vegetables in the Brassicaceae family)	GST induction potentially useful for lung cancer	(in vivo) acrylonitrile-treated male Sprague–Dawley rats with streptozotocin-induced diabetes pretreated with 20, 40, and 80 mg/kg PEITC	[76]
sulforaphane [*Brassica oleracea* L. vegetables like broccoli (var. *italica*), cabbage (var. *capitata*), cauliflower (var. *botrytis*), kale (var. *acephala*)] (isothiocyanate)	GST induction resulting in decreased acrylamide detoxification;	(in vitro) Caco-2 cells treated with either 2.5 mM acrylamide, 10 μM SFN or the combination of both for 24 h;	[37]
	induced GST A1 mRNA expression;	(in vitro) human HepG2 cells exposed to 2-amino-1-methyl-6-phenylimidazo [4,5-b]pyridine (sulforaphane 1–10 μM);	[77]
	expression of GSTP1-1 proteins increased by 3 to 5-fold	(in vitro) MCF-10F cells (human mammary epithelial cell line) treated with sulforaphane (0.5–2.0 μM)	[78]
**Organosulfur compounds**			
allyl methyl disulfide (*Allium sativum* L., garlic, and *Allium cepa* L., onion)	Induced GST activity in the forestomach, liver, small intestine, and lung	(in vivo) benzo[a]pyrene induced neoplasia of the forestomach and lung of female A/J mice	[79]
allyl methyl trisulfide (*Allium sativum* L., garlic, and *Allium cepa* L., onion)	Induced GST activity in the forestomach, liver, small intestine, and lung	(in vivo) benzo[a]pyrene induced neoplasia of the forestomach and lung of female A/J mice	[79]
diallyl sulfide (*Allium sativum* L., garlic)	increased level of alpha, mu, and pi class GSTs in the stomach of the mice	(in vivo) orally administered (25, 50, and 75 μM) to female A/J mice	[80]
diallyl disulfide (*Allium sativum* L., garlic, and *Allium cepa* L., onion)	increased GST activity in the liver, colon, jejunum, forestomach, glandular stomach, kidney, duodenum, cecum, lung, and ileum	(in vivo) Female rats from the Ru Akura colony of Sprague-Dawley-derived animals, 500 μM/kg body wt/day compound dosed by oral intubation, 5 days	[81]
diallyl sulfide (*Allium sativum* L., garlic, and *Allium cepa* L., onion)	increased GST activity in the liver, colon, jejunum, glandular stomach, cecum, and lung.	(in vivo) Female rats from the Ru Akura colony of Sprague-Dawley-derived animals, 500 µmol/kg body wt/day compound dosed by oral intubation 5 days	[81]
diallyl trisulfide (*Allium sativum* L., garlic, and *Allium cepa* L., onion)	increased GST activity in the liver, colon, jejunum, glandular stomach, kidney, duodenum, lung, and ileum (decreased in forestomach)	(in vivo) Female rats from the Ru Akura colony of Sprague-Dawley-derived animals, 500 µmol/kg body wt/day compound dosed by oral intubation, 5 days	[81]
dipropenyl disulfide (*Allium sativum* L., garlic, and *Allium cepa* L., onion)	increased GST activity in the liver, glandular stomach, duodenum, ileum, cecum, lung, and urinary bladder; increased GST activity in the liver, glandular stomach, duodenum, urinary bladder, and kidney	(in vivo) Female rats from the Ru Akura colony of Sprague-Dawley-derived animals, 500 µmol/kg body wt/day compound dosed by oral intubation, 5 days	[81]
dipropyl disulfide (*Allium sativum* L., garlic, and *Allium cepa* L., onion)	increased GST activity in the glandular stomach (no effect on liver, colon, jejunum, cecum, and lung)	(in vivo) Female rats from the Ru Akura colony of Sprague-Dawley-derived animals, 500 µmol/kg body wt/day compound dosed by oral intubation 5 days	[81]
dipropyl sulfide (*Allium sativum* L., garlic, and *Allium cepa* L., onion)	increased GST activity in the liver (no effect on colon, jejunum, glandular stomach, cecum, and lung)	(in vivo) Female rats from the Ru Akura colony of Sprague-Dawley-derived animals, 500 µmol/kg body wt/day compound dosed by oral intubation 5 days	[81]
1,2-dithiole-3-thione (*Brassicaceae* vegetables)	GST A and GST M induction via protein and mRNA expression	(in vitro) normal rat kidney (NRK-52E) proximal tubular cells incubated with 10-50 μM of 1,2-dithiole-3-thione	[82]
diallyl sulfide (DAS), diallyl disulfide (DADS), diallyl trisulfide (DATS) (*Allium* species)	increase in hepatic and forestomach GST	(in vivo) mice treated with the carcinogenic benzo(a)pyrene	[83]
dipropyl sulfide (DPS), dipropyl disulfide (DPDS), and diallyl disulfide (DADS) (*Allium* species)	stimulated GST activity	(in vivo) rat	[84]
goitrin (*Brassica oleracea* var. *gemmifera* DC., Brussels sprouts)(organosulfur compound, oxazolidinones)	increased levels of hepatic GST protein (1.4-fold), without effect on intestinal GST	(in vivo) rats fed on a goitrin-supplemented diet (200 mg/kg diet)	[85]
disulfiram (thioamide)	increased GST activity	(in vivo) liver and small intestine in female ICR/Ha mice	[50]
**Indole, indole derivatives**			
3,3′-diindolylmethane (DIM) [mechanically damaged *Brassicaceae* vegetables (broccoli, cabbage, cauliflower, Brussels sprouts)] (indole)	promoted GST expression	(in vivo) protective role of DIM (25 mg/kg b.w., p.o. in concomitant and 15 days pretreatment schedule) against doxorubicin(5 mg/kg b.w., i.p.) -induced toxicity in mice	[86]
indole-3-carbinol [result of the breakdown of the glucosinolate glucobrassicin, present in *Brassicaceae* vegetables (broccoli, cabbage, cauliflower, Brussels sprouts, kale] (indolyl alcohol)	increased GST activity;	(in vivo) liver and small intestine in female ICR/Ha mice;	[50]
	increased hepatic and intestinal GSTs by 1.9-and 1.6-fold	(in vivo) male Sprague-Dawley rats fed on an indole-3-carbinol-supplemented diet (50–500 ppm)	[87]
**Lactones**			
α-angelicalactone (*Angelica* spp.) (butenolide)	GST induction (GST A > GST M > GST P), potentially chemopreventive in the stomach, small intestine, liver, and to a lesser extent in the esophagus	(in vivo) male Wistar rats	[68]
andrographolide (*Andrographis paniculata* (Burm.f.) Nees, creat) (diterpene lactone)	induces GST gene expression by activation of the PI3K/Akt, phosphorylation of c-jun, nuclear accumulation of activator protein-1, and binding to the response element in the gene promoter region;	(in vitro) rat hepatocytes treated with 40 μM andrographolide;	[88]
	dose-dependently induced GST P protein and mRNA expression	(in vitro) rat primary hepatocytes treated with 10 or 20 μM andrographolide	[89]
α-angelicalactone (*Picea abies* (L.) H.Karst., spruce)	increased GST activity	(in vivo) liver and small intestine in female ICR/Ha mice	[50]
3-n-butyl phthalide (seed oil from *Apium graveolens* L., wild celery) (phthalide)	increased GST activity	(in vivo) female A/J mice, liver and small intestinal mucosa; 20 mg/dose every two days for a total of 3 doses	[57]
sedanolide(seed oil from *Apium graveolens* L., wild celery) (tetrahydrophthalide)	increased GST activity	(in vivo) female A/J mice, liver and small intestinal mucosa; 20 mg/dose every two days for a total of 3 doses	[57]
**Quinones**			
thymoquinone (*Nigella sativa* L., black caraway)	increased Nrf2 nuclear translocation with translation of genes for antioxidant enzymes, including GST;	(in vitro) Human neuroblastoma SH-SY5Y cells; C57/BL6 mice;	[90]
	GST was significantly induced by the high dose	(in vivo) female New Zealand White rabbits treated with thymoquinone 10 and 20 mg/kg/day orally for 8 weeks	[91]
shikonin (*Lithospermum erythrorhizon* Siebold & Zucc., purple gromwell) (naphthoquinone pigment)	increased protein and RNA expression of GST	(in vitro) primary hepatocytes isolated from Sprague-Dawley rats treated with 0–2 μM shikonin	[92]
**Stilbenoids**			
salvianolic acid B (*Salvia miltiorrhiza* Bunge, red sage)	increased GST expression	(in vitro) HepG2 cells incubated with 1 μmol/L and 10 μmol/L salvianolic acid B	[93]
resveratrol (many sources: grapes, blueberries, raspberries, mulberries, peanuts, etc.)	GSTM2 promoter activation leading to increased; GSTM2 mRNA and protein expression; GST induction potentially useful for lung cancer decreased GST activity in a concentration-dependent	(in vitro) bladder cancer cell lines, 5637 and BFTC 905;	[53]
		(in vivo) benzo(a)pyrene (BP)-induced lung carcinogenesis in male Laka mice treated with curcumin orally 60 mg/kg/body weight thrice a week + resveratrol 5.7 mg/mL thrice a week 10 days before BP injection;	[47]
		(in vitro) (colon carcinoma cell line, Caco-2)	[94]
**Others**			
myristicin [essential oil extracted from the leaves of *Petroselinum crispum* (Mill.) Fuss, parsley (allylbenzene derivative)	induction of GST in the liver and small intestinal mucosa	(in vivo) female A/J mice	[95]
curcumin (*Curcuma longa* L., turmeric) (diarylheptanoid)	GSTM2 promoter activation leading to increased GSTM2 mRNA and protein expression;	(in vitro) bladder cancer cell lines, 5637 and BFTC 905;(in vivo) benzo(a)pyrene (BP)-induced lung carcinogenesis in male Laka mice treated with curcumin orally 60 mg/kg/body weight thrice a week + resveratrol 5.7 mg/mL thrice a week 10 days before BP injection	[53]
	GST induction potentially useful for lung cancer		[47]
1-cyano-2,3-epithiopropane, 1-cyano-3,4-epithiobutane, 1-cyano-4,5-epithiopentane [*Brassicaceae* vegetables] (epithionitrile)	Inhibition of Keap1 → induction of Nrf2 → activation of ARE → induction -> induction of GST	(in vitro) rat liver RL-34 epithelial cells treated with 50 μM of epithionitrile	[49]
geniposide (fruits of *Gardenia jasminoides* J.Ellis, gardenia) (iridoid glyco-side)	increased hepatic cytosolic GST activity	(in vivo) rats treated orally with 0.1 g/kg body weight/day	[96]
folic acid (green leafy vegetables, beans, fruits,) (vitamin)	Increased expression of GST-4	(in vivo) folic acid (25 μM) treated *Caenorhabditis elegans* (a nematode used as an experimental aging model)	[97]

## 4. Glutathione-S-Transferase Inhibition by Phytochemicals

The expression of certain glutathione S-transferase isoenzymes (e.g., GSTP1) is elevated in many types of cancer and is associated with the promotion of cancer development (through apoptosis inhibition and uncontrolled cell proliferation) as well as with multidrug resistance (MDR) [6,98].

Therefore, inhibitors of GSTs might have beneficial effects as adjuvant agents in cancer treatment, mainly by reducing MDR and sensitizing cancer cells to anticancer drugs.

Phytochemicals that have demonstrated GST-inhibitory potential are summarized in Table 3.

**Table 3:** Phytochemicals with inhibitory activity on glutathione-S-transferase (GST).

Phytochemicals (Source/Chemical Subclass)	Mechanisms of Action	Type of Study	References
**Alkaloids**			
quinidine, quinine (*Cinchona officinalis* L., quinine) (cinchona alkaloid)	GST M1-1 and GST P1-1 activity inhibition	(in vitro) inhibition studies with human recombinant GSTs heterologously expressed in *Escherichia coli* (GST M1-1: IC50 of 12 μM, 17 μM; GST P1-1: IC50 1 μM, 4 μM)	[99]
piperlongumine (*Piper longum* L., long pepper) (piperidine alkaloid)	decreased level of GST P1	(in vitro) head and neck cancer cells) and in vivo model (immunoblotting)	[100]
**Phenolics (flavonoids)**			
baicalein (flavone), baicalin (glucuronide of baicalein) (root of *Scutellaria baicalensis* Georgi., Baikal skullcap)	human erythrocyte GST inbition	(in vitro) assay; IC50: 28.75, 57.50 μM	[101]
fisetin (flavonol)	GST A1-1 reversible inhibition	(in vitro) kinetic inhibition assay and in vitro (CaCo-2 cells), IC50 1.2 ± 0.1 μΜ	[102]
phloretin (found in many fruits, including apples) (dihydrochalcone) (derived from its glucoside phloridzin)	GST activity inhibition	(in vitro) human erythrocytes (IC50: 769.10 and 99.02 μM)	[101]
catechin (fruits, including peaches, berries, red grape, bananas) (flavan-3-ol)	GST P1-1 activity inhibition	(in vitro) (breast cancer cells MCF-7; IC50 = 220 μM)	[98]
myricetin [vegetables (tomatoes, etc.), fruits (oranges, etc.), nuts, berries, tea, red wine] (flavonol)	GST A1-1 enzyme activity inhibition	(in vitro) kinetic inhibition assay with recombinant hGSTA1-1; IC50 = 2.1 ± 0.2 μΜ	[103]
naringenin (common in citrus fruits) (flavanone)	decreased mRNA expression levels of GST	(in vivo) treatment with naringenin in carbon tetrachloride (CCl4)-induced liver injury in rats	[71]
**Phenolics (tannins)**			
tannic acid [*Tara spinosa* (Molina) Britton & Rose, tara]	GST S (HPGDS) inhibition (linear competitive inhibition)	(in vitro) enzyme inhibition biochemical assays (IC50 = 0.4 μM)	[26]
thonningianin A (*Thonningia sanguinea* Vahl)	inhibition of rat liver cytosolic GST activity in a non-competitive (towards and concentration dependent manner; inhibition of human GST	(in vitro) assay on rat liver cytosolic GST using 1-chloro-2,4-dinitrobenzene (CDNB) as substrate (IC50 of 1.1 microM); inhibition of human GST P1-1; IC50 of 3.6 μM	[104]
**Other phenolics**			
6-shogaol, 10-shogaol, 6-gingerol, 10-gingerol (*Zingiber officinale* Roscoe, ginger)	decreased GST P expression	(in vitro) 100 μM of 6-shogaol, 10-shogaol, 6-gingerol, and 10-gingerol treatment at 24 h in PC3R cells (docetaxel-resistant human prostate cancer cell lines)	[105]
gossypol (*Gossypium arboreum* L., cotton) (phenolic aldehyde)	enzyme activity inhibition	(in vitro) (breast cancer cells MCF-7), IC50 = 40 μM	[98]
**Other aromatic compounds**			
cinnamaldehyde (*Cinnamomum aromaticum* Nees, cinnamon tree) (aromatic alpha, beta-unsaturated aldehyde)	moderate inhibitor of GST P1-1	(in vitro) (human IGR-39 melanoma cells)	[106]
pipataline (*Guilandina major* (*Medik.*) *Small*, *grey nicker*) (benzodioxole)	GST activity inhibition	(in vitro) direct biochemical inhibition assay; IC50: 57 μM	[107]
curcumin (*Curcuma longa* L., turmeric) (diarylheptanoid)	selective inhibitor of GST P1-1;	(in vitro) (human IGR-39 melanoma cells), 96% inhibition at 25 μM;	[106]
	inhibition of GST P1-1 at the level of transcription	(in vitro) (K562 leukemia cells), 25% inhibition by 10 μM	[108]
**Terpenes/terpenoids**			
abscisic acid (a phytohormone) (sesquiterpenoid)	inhibition GST P1-1 (hpGSTP1-1) activity	(in vitro) kinetic assay using placental glutathione S-transferase; IC50: 5.3 mM	[109]
gibberellic acid (a phytohormone) (pentacyclic diterpene acid)	Inhibition of placental glutathione S-transferase P1-1 (hpGST P1-1)	(in vitro) kinetic assay using placental glutathione S-transferase; IC50: 5.0 mM	[109]
caesaldekarin J (*Caesalpinia bonduc* L.) (diterpene)	GST activity inhibition	(in vitro) direct biochemical inhibition assay; IC50: 250 μM	[107]
**Sterols**			
3a-acetoxy-13,14-seco-stigmasta-9(11),14-diene (*Guilandina major* (*Medik.*) *Small*, *grey nicker*)	GST activity inhibition	(in vitro) direct biochemical inhibition assay; IC50: 153 μM	[107]
5b,6b-epoxy-13,14-seco-stigmast-14-en-3a-ol (*Guilandina major* (*Medik.*) *Small*, *grey nicker*)	GST activity inhibition	(in vitro) direct biochemical inhibition assay; IC50: 118 μM	[107]
17-hydroxy-campes-ta-4,6-dien-3-one (*Caesalpinia bonduc* L.)	GST activity inhibition	(in vitro) direct biochemical inhibition assay; IC50: 380 μM	[107]
3-oxo-13,14-seco-stigmasta-9(11),14-diene *Guilandina major* (*Medik.*) *Small*, *grey nicker*)	GST activity inhibition	(in vitro) direct biochemical inhibition assay; IC50: 158 μM	[107]
13,14-seco-stigmas-ta5,14-dien-3a-ol (*Caesalpinia bonduc* L.)	GST activity inhibition	(in vitro) direct biochemical inhibition assay; IC50: 230 μM	[107]
13,14-seco-stigmas-ta-9(11),14-dien-3a-ol (*Guilandina major* (*Medik.*) *Small*, *grey nicker*)	GST activity inhibition	(in vitro) direct biochemical inhibition assay; IC50: 248 μM	[107]
**Others**			
phenethyl isothiocyanate (Brassicaceae vegetables) (isothiocyanate)	inhibition of hGST P1 and hGST A1	(in vitro) kinetic assay using 1-chloro-2, 4-dinitrobenzene as enzyme substrate	[46]
tetra-and hexahydro isoalpha acids (*Humulus lupulus* L., hops) (isoalpha acids)	reduced expression of glutathione-S-transferase in the liver	(in vitro) human hepatocellular carcinoma (HCC) cell lines (HepG2, Hep3B, Huh7) and in vivo in diethylnitrosamine (DEN)-induced animal model of HCC	[110]
artemisinin (*Artemisia annua* L., sweet wormwood) (sesquiterpene lactone)	GST A1-1 and GST P1-1 activity inhibition	(in vitro) inhibition studies with human recombinant GSTs heterologously expressed in *Escherichia coli* (GST A1-1: IC50 of 6 μM; GST P1-1: IC50 values of 2 μM)	[99]
oridonin (*Isodon rubescens* (Hemsl.) H.Hara) (tetracyclic diterpenoid)	downregulated expression of GST P	(in vitro) (PANC-1/Gem cells) (concentration used 40, 60 μM)	[111]
RR-α-tocopherol (nuts, seeds, vegetable oils) (prenol lipid)	GST A1-1, P1-1, M1-1 activity inhibition	(in vitro) assay with purified enzymes (GST P1-1: IC50 = 0.6 ± 0.06 μM; GST A1-1: IC50-0.9 ± 0.08 μM, GST M1-1: IC50-1.2 ± 0.06 μM; GST A2-2-IC50 3.5 ± 0.06 μM), human liver cytosol (GST M and GST A 281 ± 4 μM in) and lysate of human erythrocytes (GST: 103 ± 17 μM)_	[112]

Legend (): (type of GST enzyme or variant inhibited).

## 5. GST Activation or Inhibition-Which Interventional Strategy Is the Better Choice?

Given the functional complexity and versatility of GSTs, it remains an open question whether activation or inhibition of GST represents the more appropriate interventional strategy.

There are two main conditions in which intervention in GST activity may be beneficial: (1) exposure to xenobiotics and (2) multidrug resistance to chemotherapy resulting from high GST expression in neoplastic tissue. Based on the biological functions of GSTs in each specific condition, various approaches may be appropriate.

**Figure 1:**
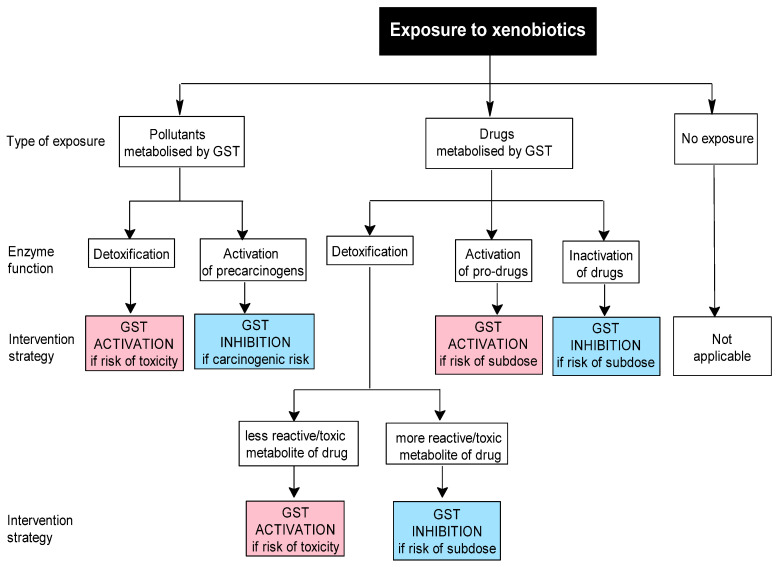
Proposed decisional algorithm for selecting a GST interventional strategy in cases of xenobiotic exposure.

**Figure 2:**
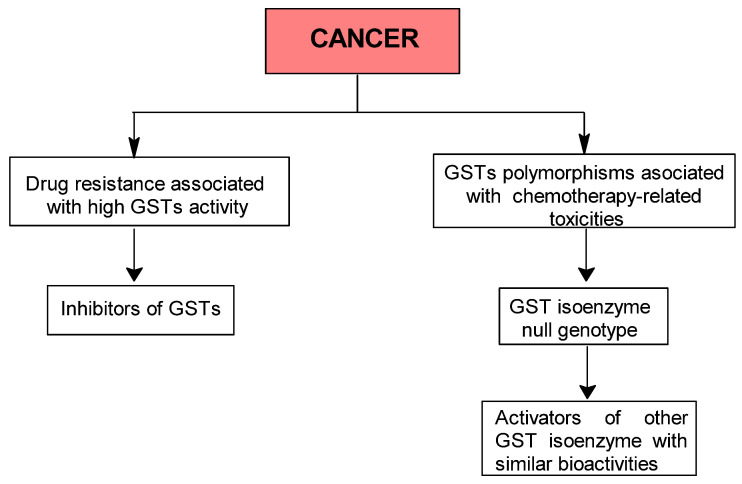
Proposed step-by-step algorithm for selecting GST activation or inhibition strategies in cancer therapy.

To clarify their relevance, we propose two step-by-step decisional algorithms (Figure 1 and Figure 2) designed to guide researchers and clinicians in selecting optimal preventive or therapeutic strategies targeting GST activity. These algorithms highlight key factors—such as genetic polymorphisms, co-exposure to xenobiotics, dietary influences, and paradoxical effects like increased toxicity or drug resistance—to support personalized decisions. For example, they show when GST induction can enhance detoxification in environmental or occupational exposures, but also when inhibition may be preferable to prevent harmful metabolite formation.

The first algorithm addresses xenobiotic exposure. If such exposure is suspected, during the first step, the investigator should establish whether it involves an environmental pollutant (e.g., bioaccumulative organochlorine pesticides) or a GST-metabolized drug, using specific approaches such as anamnesis and/or measurements of xenobiotic levels in various biological samples (Figure 1). In the second step, the investigator should evaluate the GST function in relation to the implicated xenobiotic, which may be positive (e.g., detoxification or activation of a prodrug) or negative (activation of a precarcinogen or inhibition of drug efficacy). Based on this evaluation, the investigator then decides the appropriate strategy: either activation or inhibition of GST.

In the case of xenobiotic exposure, the conjugation of GSH with xenobiotics generally produces metabolites that are less reactive or toxic and more easily excreted. In such situations, activation of GSTs is desirable if there is a risk of toxicity. Nevertheless, in certain cases, the resulting metabolites can be more reactive or toxic than their parent compounds. In these instances, the inhibition of GSTs may be more beneficial, at least in the short term, to reduce the risk of acute toxicity. For example, GSH-dependent conjugation of dihaloalkanes, such as the conjugation of dichloromethane, an industrial solvent, by GST T1, results in the formation of highly unstable metabolites (e.g., chloromethyl glutathione adducts, formaldehyde) with carcinogenic potential [113,114,115,116].

An important research trend in cancer therapy is the development of novel drugs that can suppress or circumvent the phenomenon known as multidrug resistance (MDR), which results from several mechanisms. One strategy employed by cancer cells to evade the cytotoxic effects of antitumor drugs is the overexpression of detoxification enzymes, including GSTs (Table 4), which have been shown to play key roles in cell survival and death signaling. Some GST classes (especially GST P and GST M) modulate the mitogen-activated protein (MAP) kinase pathway by directly interacting with signaling molecules like c-Jun N-terminal kinase 1 (JNK1) and apoptosis signal-regulating kinase (ASK1), or through post-translational modifications of specific proteins [117,118]. Among these, GST P1 has been identified as the isoenzyme most highly expressed in human cancerous and precancerous tissues in a screening program [119]. Interestingly, a similar mechanism underlies resistance to insecticides [120], and chloroquine in *Plasmodium falciparum* [121] and the anti-nematode activity of certain medicinal plants [122].

Several widely used anticancer drugs (e.g., alkylating agents, doxorubicin) are known substrates of GSTs. Consequently, GST inhibitors may contribute to overcoming MDR and/or enhancing cancer cell sensitivity to treatment.

**Table 4:** Examples of multidrug resistance to anticancer drugs attributable to the overexpression of GSTs.

Isoenzyme	Drug Resistance	Biological Model	
GST	melphalan	Chinese hamster ovary cells (drug-sensitive AuxB1 and multidrug-resistant CH(R)C5) in a clonogenic survival assay to assess effects of hyperthermia (41–43 degrees C), ethacrynic acid (a glutathione S-transferase inhibitor), and melphalan on cytotoxicity	[121]
	chlorambucil; cyclophosphamide; bendamustine, melphalan	lonidamine potentiating effect on nitrogen mustard alkylating agents (chlorambucil; cyclophosphamide; bendamustine, melphalan) in the systemic treatment of DB-1 human melanoma xenografts in mice	[122]
GST A1	chlorambucil	HepG2 human liver cancer cells with high levels of multidrug resistance protein 2 (MRP2), which potentiates glutathione S-transferase A1-1 (GSTA1-1)-mediated resistance to chlorambucil cytotoxicity	[123]
	chlorambucil	MCF7/WT, MCF7/VP, and MCF7/VPa human breast carcinoma cells used to study synergy between GSTA1-1 and MRP1 in resistance to chlorambucil (but not melphalan); MRP1 required to relieve product inhibition of GSTA1-1 by CHB-SG	[124]
GST M	chlorambucil	chlorambucil-resistant A2780 human ovarian carcinoma cells overexpressing GSTμ isoform to study acquired resistance to alkylating agents	[125]
GST M1	vincristine, chlorambucil	melanoma cells resistant to CHB due to GSTM1 and resistant to vincristine due to the synergy between GSTM1 and multidrug resistance protein 1; CAL1 human melanoma cells engineered to overexpress GSTM1 to study resistance to chlorambucil (via GSTM1) and vincristine (via GSTM1 synergy with MRP1)	[126]
GST P1	doxorubicin	HEp2 human carcinoma cells (parental, doxorubicin-resistant subclone HEp2A, and GSTP1-transfected) to study roles of GSTP1, P-gp, and MRP1 in doxorubicin resistance	[127]
	1,3-bis(2-chloroethyl)-1-nitrosourea (BCNU)	biopsy specimens from malignant glioma patients (astrocytoma, anaplastic astrocytoma, glioblastoma multiforme, oligodendroglioma, glioma); MGMT and GSTP1 overexpression are independently associated with BCNU resistance, greatest resistance seen with co-expression	[128]
GST-P	cyclophosphamide, adriamycin, vincristine	two chronic lymphocytic leukemia patients refractory to cyclophosphamide + adriamycin + vincristine + prednisone due to GST-Pi and GP-170 overexpression	[129]
	cyclophosphamide	GST-pi gene-transfected mice resistant to cyclophosphamide due to bone marrow chemoprotection.	[130]
	cisplatin	haloenol lactone derivative potentiates cisplatin-induced cytotoxicity in UOK130 human renal tumor cells by inhibiting GST-pi and MRP1-3	[131]
	cisplatin, melphalan, chlorambucil	multiple drug-resistant UOK130 renal tumor cells with selective GST-pi over-expression (via cisplatin escalation or GST-pi cDNA transfection); resistance reversed by haloenol lactone GST-pi inhibitor	[132]
GSTP1-1	adriamycin, cisplatin, and alkylating agents such as melphalan and 4-hydroxyperoxycyclophosphamide	human cholangiocarcinoma cells: GSTP1-1 antisense transfection decreases intracellular GSTP1-1 levels and increases sensitivity to adriamycin, cisplatin, melphalan, and 4-hydroxyperoxycyclophosphamide; C16C2, a GSTP1-1-specific inhibitor, also reduces resistance	[133]
	thiotepa	GST-P1-1-transfected human MCF-7 breast cancer cells: overexpression of GST-P1-1 increases formation and efflux of monoglutathionylthiotepa, reducing thiotepa cytotoxicity; inhibition of GST, GSH synthesis, or glutathione conjugate efflux (e.g., with ethacrynic acid, BSO, probenecid, or verapamil) decreases conjugate formation or transport and enhances cytotoxicity	[134]

Genetic polymorphisms in GSTs have been linked to an increased risk of various types of cancers. For example, the GSTT1 null genotype is associated with lymphoma, breast, prostate, and oral cancer, as well as a subtype of basal cell carcinoma characterized by a tendency to develop multiple primary tumors in clusters [4,125,126,127]. Similarly, the GSTM1 null genotype has been linked to breast, prostate, liver, and oral cancers [125,126,127,128]. The reduced detoxication capacity associated with these genotypes is believed to contribute to their increased susceptibility to malignancies [129]. Additionally, there is a complex interplay among dietary factors and gene polymorphisms that may further influence cancer risk [130]. For instance, a high intake of red meat has been associated with a greater likelihood of developing colorectal cancer in individuals carrying the GSTM1 null genotype [130].

A potential preventive or therapeutic strategy for individuals with GST null genotypes, which are associated with a higher risk of cancer, is to enhance the activity of other functional GST isoenzymes with similar roles to those that are absent. For instance, as previously mentioned, GST M1 null genotypes are associated with a high risk of diverse malignancies [128]. One of the important functions of GST M1 is to inhibit stress kinases (e.g., JNK, ASK1) through protein–protein interactions [2,14,22]. These kinases signaling pathways, which are activated by various stressors (oxidant agents, UV, proinflammatory cytokines, heat shock, etc.), play important regulatory roles in cell death, immune response, inflammation, and hormesis [131,132,133]. Other GSTs also displayed similar properties, and their activation by an array of phytochemicals may compensate for the absence of GST M1-1. As an example, GST P1-1 binds to JNK, and its overexpression results in decreased JNK activity, thus protecting against cell death [15]; GST A1-1 binds to JNK, and its overexpression significantly reduced activation of c-Jun, suggesting a protective role for GSTA1-1 in JNK-associated apoptosis [13]. JNK activity is also decreased in hGSTA2-2-overexpressing K562 cells, protecting them against H_2_O_2_-induced apoptosis. Transfection of *mGsta4* into HL-60 cells inhibits JNK-mediated signaling and consequently exerts an antiapoptotic effect [134]. Thus, in individuals with GST M1 null genotypes, the activation or overexpression of GST P1-1, GST A1-1, GST A2-2, or GST A4 may potentially compensate for the secondary detrimental effects associated with this genetic deficiency.

Given that GST overexpression contributes to multidrug resistance and tumor progression, while GST null genotypes are linked to increased susceptibility to various cancer types, we propose that targeting GST with specific inhibitors or modulators represents a rational adjuvant strategy to complement conventional therapies.

Thus, the purpose of the second algorithm is to guide the investigator in identifying a potential GST-based interventional strategy in cancer patients. In the first step, the investigator should determine whether the patient has a multidrug-resistance phenotype associated with GST overexpression or carries a GST null genotype. Depending on the result of the first step, inhibition or activation of GSTs can then be selected (Figure 2).

Moreover, limited detoxification capacity can be partly offset by dietary or nutraceutical inducers like sulforaphane, an isothiocyanate present in cruciferous vegetables. Sulforaphane has been shown to increase the levels of several functional GSTs, including GSTP1, GSTM2, and GSTA1 [5,135]. In addition, the Nrf2 signaling pathway-an important regulator of phase II detoxification enzymes, including several GSTs-can be activated by several phytochemicals and synthetic compounds [136,137].

Nevertheless, it is important to note that these algorithms are intended as a starting point for systematic, evidence-based decision making, and their clinical applicability should be validated through further studies. In practice, nutraceutical interventions may not be straightforward, especially in cases involving phytochemicals with dual activity or contradictory evidence, where both GST activation and inhibition have been reported. Examples include compounds such as naringenin [83,84,138] or curcumin [100,101,139], for which both activatory and inhibitory effects have been documented.

## 6. Conclusions

This study explores emerging trends in how phytochemicals can modulate GSTs, key detoxification enzymes with pleiotropic functions. A substantial share of phytochemicals with GST-modulating potential is found among polyphenolic classes, such as flavonoids (naringenin, quercetin, and resveratrol) and phenolic acids (ferulic and gallic acid) [140,141], as well as terpenoids (e.g., curcumin, 6-shogaol) or isothiocyanates (e.g., sulforaphane) [5,136]. The activation effects predominate, mainly due to the ability of these compounds to induce phase II detoxifying enzymes, via the Nrf2-ARE pathway that guards against xenobiotic toxicity and oxidative stress [5,136]. On the other hand, GST inhibitory effects are exerted by a smaller, but clinically relevant group of compounds, with notable implications in tumoral tissues where GST overexpression is associated with drug resistance. For example, GSTP1, a GST isoenzyme commonly overexpressed in breast, colon, and lung cancers, can be selectively inhibited by chalcones and flavonoids [2,118,138].

Several issues require attention before these strategies can be considered suitable for clinical application. Due to overlapping—although distinct—substrate specificities of GST isoenzymes, modulating one can have inadvertent effects on others [5]. For example, inhibiting GSTP1 may concurrently resensitize neoplastic cells and reduce antioxidant protection in healthy tissues [2]. Furthermore, the risk of off-target effects is augmented by a deficit of accurate isoenzyme selectivity that characterizes several phytochemicals [141,142]. Another aspect concerns the disparities between the bioavailability and metabolic pathways of these compounds in in vitro models versus humans, as well as the fact that concentrations efficient in cell cultures may not be achievable or safe in vivo [143,144].

Further complexity is added by interindividual variability. Phytochemical metabolism and its impact on GST activity can be influenced by genetic polymorphisms like GSTM1 or GSTT1 null genotypes, coexisting conditions, age, or particulars of the gut microbiota [5,30,145]. Additionally, potential interactions with conventional therapies or other dietary components, dual or dose-dependent effects, and the presence of antagonistic or redundant compounds in crude extracts must be carefully considered [140,141].

A nuanced understanding of when to activate or inhibit GSTs—a question at the heart of this review—is essential for making compelling and reliable therapeutic decisions. Whereas enhancing GST activity can boost the body’s detoxification of harmful xenobiotics, inhibiting overexpressed GST isoenzymes may serve as an adjuvant during chemotherapy for multidrug-resistant cancers characterized by high GST expression in neoplastic cells [2,118]. Furthermore, the same compound can sometimes exert differing effects depending on the dose, metabolic context, or tissue distribution [140,142]

To navigate these challenges, we propose decisional algorithms as conceptual frameworks to guide whether GST activation or inhibition should be pursued in a given context. However, these algorithms remain theoretical until they are systematically validated in experimental and clinical settings [146].

Therefore, while GST-modulating phytochemicals have potential as preventive or adjuvant strategies, further comprehensive studies and robust investigations across diverse populations are required to deepen our understanding of the complex biological roles of GSTs. Emerging tools such as organoids or patient-derived xenografts may better predict human responses and clarify isoenzyme selectivity and off-target effects [142].

In conclusion, whether to activate or inhibit GSTs depends on the specific physiological or pathological context. Phytochemicals may be suitable for both strategies, but their safe and efficient use demands a solid understanding of GST biology, careful patient stratification, and rigorous experimental validation.

## Data Availability

Not applicable.

## Abbreviations

ARE—antioxidant responsive element; GPx—glutathione reductase; DIM—3,3′-diindolylmethane; GR—glutathione reductase; GST—glutathione-S-transferase; GSTM2—GST mu2; HPGDS—hematopoietic prostaglandin D synthase; Keap1—Kelch-like ECH-associated protein 1; Nrf2—NF-E2 p45-related factor 2; PEITC—phenethyl isothiocyanate; SOD—superoxide dismutase.

## References

[1] Jakoby W.B. (1978). The glutathione S-transferases: A group of multifunctional detoxification proteins. Adv. Enzymol. Relat. Areas Mol. Biol..

[2] Townsend D.M., Tew K.D. (2003). The role of glutathione-S-transferase in anti-cancer drug resistance. Oncogene.

[3] Singh R.R., Reindl K.M. (2021). Glutathione S-Transferases in Cancer. Antioxidants.

[4] Strange R.C., Spiteri M.A., Ramachandran S., Fryer A.A. (2001). Glutathione-S-transferase family of enzymes. Mutat. Res..

[5] Hayes J.D., Flanagan J.U., Jowsey I.R. (2005). Glutathione transferases. Annu. Rev. Pharmacol. Toxicol..

[6] Alnasser S.M. (2025). The role of glutathione S-transferases in human disease pathogenesis and their current inhibitors. Genes Dis..

[7] Board P.G., Anders M.W., Sies H., Packer L. (2005). Human Glutathione Transferase Zeta. Gluthione Transferases and Gamma-Glutamyl Transpeptidases.

[8] Bocedi A., Noce A., Marrone G., Noce G., Cattani G., Gambardella G., Di Lauro M., Di Daniele N., Ricci G. (2019). Glutathione Transferase P1-1 an Enzyme Useful in Biomedicine and as Biomarker in Clinical Practice and in Environmental Pollution. Nutrients.

[9] Maeda A., Crabb J.W., Palczewski K. (2005). Microsomal glutathione S-transferase 1 in the retinal pigment epithelium: Protection against oxidative stress and a potential role in aging. Biochemistry.

[10] Zager R.A., Johnson A.C.M. (2022). Early loss of glutathione-s-transferase (GST) activity during diverse forms of acute renal tubular injury. Physiol. Rep..

[11] Krishna Chandran A.M., Christina H., Das S., Mumbrekar K.D., Satish Rao B.S. (2019). Neuroprotective role of naringenin against methylmercury induced cognitive impairment and mitochondrial damage in a mouse model. Environ. Toxicol. Pharmacol..

[12] Coles B.F., Kadlubar F.F. (2005). Human alpha class glutathione S-transferases: Genetic polymorphism, expression, and susceptibility to disease. Methods Enzymol..

[13] Romero L., Andrews K., Ng L., O’Rourke K., Maslen A., Kirby G. (2006). Human GSTA1-1 reduces c-Jun N-terminal kinase signalling and apoptosis in Caco-2 cells. Biochem. J..

[14] Ryoo K., Huh S.-H., Lee Y.H., Yoon K.W., Cho S.-G., Choi E.-J. (2004). Negative regulation of MEKK1-induced signaling by glutathione S-transferase Mu. J. Biol. Chem..

[15] Adler V., Yin Z., Fuchs S.Y., Benezra M., Rosario L., Tew K.D., Pincus M.R., Sardana M., Henderson C.J., Wolf C.R. (1999). Regulation of JNK signaling by GSTp. EMBO J..

[16] Dunna N.R., Vure S., Sailaja K., Surekha D., Raghunadharao D., Rajappa S., Vishnupriya S. (2013). Deletion of GSTM1 and T1 genes as a risk factor for development of acute leukemia. Asian Pac. J. Cancer Prev..

[17] Hayes J.D., Pulford D.J. (1995). The glutathione S-transferase supergene family: Regulation of GST and the contribution of the isoenzymes to cancer chemoprotection and drug resistance. Crit. Rev. Biochem. Mol. Biol..

[18] Yan F.-X., Langub M.C., Ihnen M.A., Hornung C., Juronen E., Rayens M.K., Cai W.-M., Wedlund P.J., Fanti P. (2003). CYP2D6, GST-M1 and GST-T1 enzymes: Expression in parathyroid gland and association with the parathyroid hormone concentration during early renal replacement therapy. Br. J. Clin. Pharmacol..

[19] Bogaards J.J., Venekamp J.C., van Bladeren P.J. (1997). Stereoselective conjugation of prostaglandin A2 and prostaglandin J2 with glutathione, catalyzed by the human glutathione S-transferases A1-1, A2-2, M1a-1a, and P1-1. Chem. Res. Toxicol..

[20] Brunnström A., Hamberg M., Griffiths W.J., Mannervik B., Claesson H.-E. (2011). Biosynthesis of 14,15-hepoxilins in human l1236 Hodgkin lymphoma cells and eosinophils. Lipids.

[21] Kolm R.H., Danielson U.H., Zhang Y., Talalay P., Mannervik B. (1995). Isothiocyanates as substrates for human glutathione transferases: Structure-activity studies. Biochem. J..

[22] Cho S.G., Lee Y.H., Park H.S., Ryoo K., Kang K.W., Park J., Eom S.J., Kim M.J., Chang T.S., Choi S.Y. (2001). Glutathione S-transferase mu modulates the stress-activated signals by suppressing apoptosis signal-regulating kinase 1. J. Biol. Chem..

[23] Uhlen M., Fagerberg L., Hallstrom B.M., Lindskog C., Oksvold P., Mardinoglu A., Sivertsson A., Kampf C., Sjostedt E., Asplund A. (2015). Proteomics. Tissue-based map of the human proteome. Science.

[24] Dalli J., Vlasakov I., Riley I.R., Rodriguez A.R., Spur B.W., Petasis N.A., Chiang N., Serhan C.N. (2016). Maresin conjugates in tissue regeneration biosynthesis enzymes in human macrophages. Proc. Natl. Acad. Sci. USA.

[25] Thier R., Wiebel F.A., Hinkel A., Burger A., Brüning T., Morgenroth K., Senge T., Wilhelm M., Schulz T.G. (1998). Species differences in the glutathione transferase GSTT1-1 activity towards the model substrates methyl chloride and dichloromethane in liver and kidney. Arch. Toxicol..

[26] Mazari A.M.A., Hegazy U.M., Mannervik B. (2015). Identification of new inhibitors for human hematopoietic prostaglandin D2 synthase among FDA-approved drugs and other compounds. Chem. Biol. Interact..

[27] Kanaoka Y., Fujimori K., Kikuno R., Sakaguchi Y., Urade Y., Hayaishi O. (2000). Structure and chromosomal localization of human and mouse genes for hematopoietic prostaglandin D synthase. Conservation of the ancestral genomic structure of sigma-class glutathione S-transferase. Eur. J. Biochem..

[28] Board P.G., Coggan M., Chelvanayagam G., Easteal S., Jermiin L.S., Schulte G.K., Danley D.E., Hoth L.R., Griffor M.C., Kamath A.V. (2000). Identification, Characterization, and Crystal Structure of the Omega Class Glutathione Transferases. J. Biol. Chem..

[29] Board P.G. (2011). The omega-class glutathione transferases: Structure, function, and genetics. Drug Metab. Rev..

[30] Whitbread A.K., Tetlow N., Eyre H.J., Sutherland G.R., Board P.G. (2003). Characterization of the human Omega class glutathione transferase genes and associated polymorphisms. Pharmacogenetics.

[31] Morgenstern R., Zhang J., Johansson K. (2011). Microsomal glutathione transferase 1: Mechanism and functional roles. Drug Metab. Rev..

[32] Ji Y., Bennett B.M. (2006). Biotransformation of glyceryl trinitrate by rat hepatic microsomal glutathione S-transferase 1. J. Pharmacol. Exp. Ther..

[33] Jakobsson P.-J., Mancini J.A., Ford-Hutchinson A.W. (1996). Identification and characterization of a novel human microsomal glutathione S-transferase with leukotriene C4 synthase activity and significant sequence identity to 5-lipoxygenase-activating protein and leukotriene C4 synthase. J. Biol. Chem..

[34] Jakobsson P.-J., Mancini J.A., Riendeau D., Ford-Hutchinson A.W. (1997). Identification and characterization of a novel microsomal enzyme with glutathione-dependent transferase and peroxidase activities. J. Biol. Chem..

[35] Jakobsson P.J., Thorén S., Morgenstern R., Samuelsson B. (1999). Identification of human prostaglandin E synthase: A microsomal, glutathione-dependent, inducible enzyme, constituting a potential novel drug target. Proc. Natl. Acad. Sci. USA.

[36] Miller D.K., Gillard J.W., Vickers P.J., Sadowski S., Léveillé C., Mancini J.A., Charleson P., Dixon R.A.F., Ford-Hutchinson A.W., Fortin R. (1990). Identification and isolation of a membrane protein necessary for leukotriene production. Nature.

[37] Pernice R., Hauder J., Koehler P., Vitaglione P., Fogliano V., Somoza V. (2009). Effect of sulforaphane on glutathione-adduct formation and on glutathione_S_transferase-dependent detoxification of acrylamide in Caco-2 cells. Mol. Nutr. Food Res..

[38] Ruscoe J.E., Rosario L.A., Wang T., Gaté L., Arifoglu P., Wolf C.R., Henderson C.J., Ronai Z., Tew K.D. (2001). Pharmacologic or genetic manipulation of glutathione S-transferase P1-1 (GSTpi) influences cell proliferation pathways. J. Pharmacol. Exp. Ther..

[39] Oakley A. (2011). Glutathione transferases: A structural perspective. Drug Metab. Rev..

[40] Elmileegy I.M.H., Waly H.S.A., Alghriany A.A.I., Abou Khalil N.S., Mahmoud S.M.M., Negm E.A. (2023). Gallic acid rescues uranyl acetate induced-hepatic dysfunction in rats by its antioxidant and cytoprotective potentials. BMC Complement. Med. Ther..

[41] Figueroa-González G., Quintas-Granados L.I., Reyes-Hernández O.D., Caballero-Florán I.H., Peña-Corona S.I., Cortés H., Leyva-Gómez G., Habtemariam S., Sharifi-Rad J. (2024). Review of the anticancer properties of 6-shogaol: Mechanisms of action in cancer cells and future research opportunities. Food Sci. Nutr..

[42] Louka P., Ferreira N., Sophocleous A. (2025). Sulforaphane’s Role in Osteosarcoma Treatment: A Systematic Review and Meta-Analysis of Preclinical Studies. Biomedicines.

[43] Hadidi M., Liñán-Atero R., Tarahi M., Christodoulou M.C., Aghababaei F. (2024). The Potential Health Benefits of Gallic Acid: Therapeutic and Food Applications. Antioxidants.

[44] Shih P.-H., Yeh C.-T., Yen G.-C. (2007). Anthocyanins induce the activation of phase II enzymes through the antioxidant response element pathway against oxidative stress-induced apoptosis. J. Agric. Food Chem..

[45] Abdull Razis A.F., De Nicola G.R., Pagnotta E., Iori R., Ioannides C. (2012). 4-Methylsulfanyl-3-butenyl isothiocyanate derived from glucoraphasatin is a potent inducer of rat hepatic phase II enzymes and a potential chemopreventive agent. Arch. Toxicol..

[46] Kumari V., Dyba M.A., Holland R.J., Liang Y.-H., Singh S.V., Ji X. (2016). Irreversible Inhibition of Glutathione S-Transferase by Phenethyl Isothiocyanate (PEITC), a Dietary Cancer Chemopreventive Phytochemical. PLoS ONE.

[47] Liu Y., Wu Y.-M., Yu Y., Cao C.-S., Zhang J.-H., Li K., Zhang P.-Y. (2015). Curcumin and resveratrol in combination modulate drug-metabolizing enzymes as well as antioxidant indices during lung carcinogenesis in mice. Hum. Exp. Toxicol..

[48] Oyagbemi A.A., Omobowale O.T., Asenuga E.R., Akinleye A.S., Ogunsanwo R.O., Saba A.B. (2016). Cyclophosphamide-induced Hepatotoxicity in Wistar Rats: The Modulatory Role of Gallic Acid as a Hepatoprotective and Chemopreventive Phytochemical. Int. J. Prev. Med..

[49] Kelleher M.O., McMahon M., Eggleston I.M., Dixon M.J., Taguchi K., Yamamoto M., Hayes J.D. (2009). 1-Cyano-2,3-epithiopropane is a novel plant-derived chemopreventive agent which induces cytoprotective genes that afford resistance against the genotoxic alpha,beta-unsaturated aldehyde acrolein. Carcinogenesis.

[50] Sparnins V.L., Venegas P.L., Wattenberg L.W. (1982). Glutathione S-transferase activity: Enhancement by compounds inhibiting chemical carcinogenesis and by dietary constituents. J. Natl. Cancer Inst..

[51] Kubínová R., Machala M., Minksová K., Neca J., Suchý V. (2001). Chemoprotective activity of boldine: Modulation of drug-metabolizing enzymes. Pharmazie.

[52] Ji L., Liu T., Chen Y., Wang Z. (2009). Protective mechanisms of N-acetyl-cysteine against pyrrolizidine alkaloid clivorine-induced hepatotoxicity. J. Cell. Biochem..

[53] Shen C.-H., Wu J.-Y., Wang S.-C., Wang C.-H., Hong C.-T., Liu P.-Y., Wu S.-R., Liu Y.-W. (2022). The suppressive role of phytochemical-induced glutathione S-transferase Mu 2 in human urothelial carcinoma cells. Biomed. Pharmacother..

[54] Kelly V.P., Ellis E.M., Manson M.M., Chanas S.A., Moffat G.J., McLeod R., Judah D.J., Neal G.E., Hayes J.D. (2000). Chemoprevention of aflatoxin B1 hepatocarcinogenesis by coumarin, a natural benzopyrone that is a potent inducer of aflatoxin B1-aldehyde reductase, the glutathione S-transferase A5 and P1 subunits, and NAD(P)H: Quinone oxidoreductase in rat liver. Cancer Res..

[55] Thuong P.T., Pokharel Y.R., Lee M.Y., Kim S.K., Bae K., Su N.D., Oh W.K., Kang K.W. (2009). Dual anti-oxidative effects of fraxetin isolated from Fraxinus rhinchophylla. Biol. Pharm. Bull..

[56] Nagpal I., Abraham S.K. (2017). Ameliorative effects of gallic acid, quercetin and limonene on urethane-induced genotoxicity and oxidative stress in Drosophila melanogaster. Toxicol. Mech. Methods.

[57] Zheng G.Q., Kenney P.M., Zhang J., Lam L.K. (1993). Chemoprevention of benzo[a]pyrene-induced forestomach cancer in mice by natural phthalides from celery seed oil. Nutr. Cancer.

[58] Murali R., Karthikeyan A., Saravanan R. (2013). Protective effects of D-limonene on lipid peroxidation and antioxidant enzymes in streptozotocin-induced diabetic rats. Basic Clin. Pharmacol. Toxicol..

[59] Younis N.S., Abduldaium M.S., Mohamed M.E. (2020). Protective Effect of Geraniol on Oxidative, Inflammatory and Apoptotic Alterations in Isoproterenol-Induced Cardiotoxicity: Role of the Keap1/Nrf2/HO-1 and PI3K/Akt/mTOR Pathways. Antioxidants.

[60] Yovas A., Stanely S.P., Issac R., Ponnian S.M.P. (2023). β-caryophyllene blocks reactive oxygen species-mediated hyperlipidemia in isoproterenol-induced myocardial infarcted rats. Eur. J. Pharmacol..

[61] Zheng G.Q., Kenney P.M., Lam L.K. (1992). Sesquiterpenes from clove (Eugenia caryophyllata) as potential anticarcinogenic agents. J. Nat. Prod..

[62] Sultana S., Alam A., Khan N., Sharma S. (2003). Inhibition of benzoyl peroxide and ultraviolet-B radiation induced oxidative stress and tumor promotion markers by cycloartenol in murine skin. Redox Rep..

[63] Huber W.W., Teitel C.H., Coles B.F., King R.S., Wiese F.W., Kaderlik K.R., Casciano D.A., Shaddock J.G., Mulder G.J., Ilett K.F. (2004). Potential chemoprotective effects of the coffee components kahweol and cafestol palmitates via modification of hepatic N-acetyltransferase and glutathione S-transferase activities. Environ. Mol. Mutagen..

[64] Huber W.W., Prustomersky S., Delbanco E., Uhl M., Scharf G., Turesky R.J., Thier R., Schulte-Hermann R. (2002). Enhancement of the chemoprotective enzymes glucuronosyl transferase and glutathione transferase in specific organs of the rat by the coffee components kahweol and cafestol. Arch. Toxicol..

[65] Saleem M., Alam A., Arifin S., Shah M.S., Ahmed B., Sultana S. (2001). Lupeol, a triterpene, inhibits early responses of tumor promotion induced by benzoyl peroxide in murine skin. Pharmacol. Res..

[66] Asha R., Gayathri Devi V., Abraham A. (2016). Lupeol, a pentacyclic triterpenoid isolated from Vernonia cinerea attenuate selenite induced cataract formation in Sprague Dawley rat pups. Chem. Biol. Interact..

[67] Perez J.L., Jayaprakasha G.K., Cadena A., Martinez E., Ahmad H., Patil B.S. (2010). In Vivo induction of phase II detoxifying enzymes, glutathione transferase and quinone reductase by citrus triterpenoids. BMC Complement. Altern. Med..

[68] Nijhoff W.A., Bosboom M.A., Smidt M.H., Peters W.H. (1995). Enhancement of rat hepatic and gastrointestinal glutathione and glutathione S-transferases by alpha-angelicalactone and flavone. Carcinogenesis.

[69] Wu Z., Li C., Li Q., Li J., Lu X. (2020). Puerarin alleviates cisplatin-induced acute renal damage and upregulates microRNA-31-related signaling. Exp. Ther. Med..

[70] Rajappa R., Sireesh D., Salai M.B., Ramkumar K.M., Sarvajayakesavulu S., Madhunapantula S. (2018). V Treatment With Naringenin Elevates the Activity of Transcription Factor Nrf2 to Protect Pancreatic β-Cells From Streptozotocin-Induced Diabetes In Vitro and In Vivo. Front. Pharmacol..

[71] Esmaeili M.A., Alilou M. (2014). Naringenin attenuates CCl4-induced hepatic inflammation by the activation of an Nrf2-mediated pathway in rats. Clin. Exp. Pharmacol. Physiol..

[72] Issac P.K., Karan R., Guru A., Pachaiappan R., Arasu M.V., Al-Dhabi N.A., Choi K.C., Harikrishnan R., Raj J.A. (2021). Insulin signaling pathway assessment by enhancing antioxidant activity due to morin using In Vitro rat skeletal muscle L6 myotubes cells. Mol. Biol. Rep..

[73] Ajeigbe O.F., Maruf O.R., Anyebe D.A., Opafunso I.T., Ajayi B.O., Farombi E.O. (2022). 6-shogaol suppresses AOM/DSS-mediated colorectal adenoma through its antioxidant and anti-inflammatory effects in mice. J. Food Biochem..

[74] Bogaards J.J.P., Van Ommen B., Falke H.E., Willems M.I., Van Bladeren P.J. (1990). Glutathione S-transferase subunit induction patterns of Brussels sprouts, allyl isothiocyanate and goitrin in rat liver and small intestinal mucosa: A new approach for the identification of inducing xenobiotics. Food Chem. Toxicol..

[75] Satoh K., Yamakawa D., Kasai K., Hatayama I. (2023). Vibratome technique revealed initial carcinogenic changes that induce GST-P^+^ single hepatocytes and minifoci in rat liver. Anal. Biochem..

[76] Li F., Dong Y., Lu R., Yang B., Wang S., Xing G., Jiang Y. (2021). Susceptibility to the acute toxicity of acrylonitrile in streptozotocin-induced diabetic rats: Protective effect of phenethyl isothiocyanate, a phytochemical CYP2E1 inhibitor. Drug Chem. Toxicol..

[77] Bacon J.R., Williamson G., Garner R.C., Lappin G., Langouët S., Bao Y. (2003). Sulforaphane and quercetin modulate PhIP-DNA adduct formation in human HepG2 cells and hepatocytes. Carcinogenesis.

[78] Singletary K., MacDonald C. (2000). Inhibition of benzo[a]pyrene-and 1,6-dinitropyrene-DNA adduct formation in human mammary epithelial cells bydibenzoylmethane and sulforaphane. Cancer Lett..

[79] Sparnins V.L., Barany G., Wattenberg L.W. (1988). Effects of organosulfur compounds from garlic and onions on benzo[a]pyrene-induced neoplasia and glutathione S-transferase activity in the mouse. Carcinogenesis.

[80] Maurya A.K., Singh S. (1991). V Differential induction of glutathione transferase isoenzymes of mice stomach by diallyl sulfide, a naturally occurring anticarcinogen. Cancer Lett..

[81] Munday R., Munday C.M. (2001). Relative activities of organosulfur compounds derived from onions and garlic in increasing tissue activities of quinone reductase and glutathione transferase in rat tissues. Nutr. Cancer.

[82] Zhu H., Zhang L., Amin A.R., Li Y. (2008). Coordinated upregulation of a series of endogenous antioxidants and phase 2 enzymes as a novel strategy for protecting renal tubular cells from oxidative and electrophilic stress. Exp. Biol. Med..

[83] Srivastava S.K., Hu X., Xia H., Zaren H.A., Chatterjee M.L., Agarwal R., Singh S.V. (1997). Mechanism of differential efficacy of garlic organosulfides in preventing benzo(a)pyrene-induced cancer in mice. Cancer Lett..

[84] Siess M.H., Le Bon A.M., Canivenc-Lavier M.C., Suschetet M. (1997). Modification of hepatic drug-metabolizing enzymes in rats treated with alkyl sulfides. Cancer Lett..

[85] Kelley M.K., Bjeldanes L.F. (1995). Modulation of glutathione S-transferase activity and isozyme pattern in liver and small intestine of rats fed goitrin- and T3-supplemented diets. Food Chem. Toxicol..

[86] Hajra S., Basu A., Singha Roy S., Patra A.R., Bhattacharya S. (2017). Attenuation of doxorubicin-induced cardiotoxicity and genotoxicity by an indole-based natural compound 3,3’-diindolylmethane (DIM) through activation of Nrf2/ARE signaling pathways and inhibiting apoptosis. Free Radic. Res..

[87] Bradfield C.A., Bjeldanes L.F. (1984). Effect of dietary indole-3-carbinol on intestinal and hepatic monooxygenase, glutathione S-transferase and epoxide hydrolase activities in the rat. Food Chem. Toxicol..

[88] Lu C.-Y., Li C.-C., Lii C.-K., Yao H.-T., Liu K.-L., Tsai C.-W., Chen H.-W. (2011). Andrographolide-induced pi class of glutathione S-transferase gene expression via PI3K/Akt pathway in rat primary hepatocytes. Food Chem. Toxicol..

[89] Chang K.-T., Lii C.-K., Tsai C.-W., Yang A.-J., Chen H.-W. (2008). Modulation of the expression of the pi class of glutathione S-transferase by Andrographis paniculata extracts and andrographolide. Food Chem. Toxicol..

[90] Dong J., Zhang X., Wang S., Xu C., Gao M., Liu S., Li X., Cheng N., Han Y., Wang X. (2020). Thymoquinone Prevents Dopaminergic Neurodegeneration by Attenuating Oxidative Stress Via the Nrf2/ARE Pathway. Front. Pharmacol..

[91] Elbarbry F., Ragheb A., Marfleet T., Shoker A. (2012). Modulation of hepatic drug metabolizing enzymes by dietary doses of thymoquinone in female New Zealand White rabbits. Phytother. Res..

[92] Huang C.-S., Chen H.-W., Lin T.-Y., Lin A.-H., Lii C.-K. (2018). Shikonin upregulates the expression of drug-metabolizing enzymes and drug transporters in primary rat hepatocytes. J. Ethnopharmacol..

[93] Wang Q.-L., Wu Q., Tao Y.-Y., Liu C.-H., El-Nezami H. (2011). Salvianolic acid B modulates the expression of drug-metabolizing enzymes in HepG2 cells. Hepatobiliary Pancreat. Dis. Int..

[94] El-Readi M.Z., Eid S., Abdelghany A.A., Al-Amoudi H.S., Efferth T., Wink M. (2019). Resveratrol mediated cancer cell apoptosis, and modulation of multidrug resistance proteins and metabolic enzymes. Phytomedicine.

[95] Zheng G.Q., Kenney P.M., Zhang J., Lam L.K. (1992). Inhibition of benzo[a]pyrene-induced tumorigenesis by myristicin, a volatile aroma constituent of parsley leaf oil. Carcinogenesis.

[96] Kang J.J., Wang H.W., Liu T.Y., Chen Y.C., Ueng T.H. (1997). Modulation of cytochrome P-450-dependent monooxygenases, glutathione and glutathione S-transferase in rat liver by geniposide from Gardenia jasminoides. Food Chem. Toxicol..

[97] Rathor L., Akhoon B.A., Pandey S., Srivastava S., Pandey R. (2015). Folic acid supplementation at lower doses increases oxidative stress resistance and longevity in Caenorhabditis elegans. Age.

[98] Guneidy R.A., Shokeer A., Saleh N.S.-E., Zaki E.R. (2023). Inhibition of human glutathione transferase by catechin and gossypol: Comparative structural analysis by kinetic properties, molecular docking and their efficacy on the viability of human MCF-7 cells. J. Biochem..

[99] Mukanganyama S., Widersten M., Naik Y.S., Mannervik B., Hasler J.A. (2002). Inhibition of glutathione S-transferases by antimalarial drugs possible implications for circumventing anticancer drug resistance. Int. J. Cancer.

[100] Roh J.-L., Kim E.H., Park J.Y., Kim J.W., Kwon M., Lee B.-H. (2014). Piperlongumine selectively kills cancer cells and increases cisplatin antitumor activity in head and neck cancer. Oncotarget.

[101] Aksoy M., Küfrevioglu I. (2018). Inhibition of human erythrocyte glutathione S-transferase by some flavonoid derivatives. Toxin Rev..

[102] Alqarni M.H., Foudah A.I., Muharram M.M., Labrou N.E. (2021). The Interaction of the Flavonoid Fisetin with Human Glutathione Transferase A1-1. Metabolites.

[103] Alqarni M.H., Foudah A.I., Muharram M.M., Alam A., Labrou N.E. (2022). Myricetin as a Potential Adjuvant in Chemotherapy: Studies on the Inhibition of Human Glutathione Transferase A1-1. Biomolecules.

[104] Gyamfi M.A., Ohtani I.I., Shinno E., Aniya Y. (2004). Inhibition of glutathione S-transferases by thonningianin A, isolated from the African medicinal herb, Thonningia sanguinea, In Vitro. Food Chem. Toxicol..

[105] Liu C.-M., Kao C.-L., Tseng Y.-T., Lo Y.-C., Chen C.-Y. (2017). Ginger Phytochemicals Inhibit Cell Growth and Modulate Drug Resistance Factors in Docetaxel Resistant Prostate Cancer Cell. Molecules.

[106] Iersel M.L., Ploemen J.P., Struik I., van Amersfoort C., Keyzer A.E., Schefferlie J.G., van Bladeren P.J. (1996). Inhibition of glutathione S-transferase activity in human melanoma cells by alpha,beta-unsaturated carbonyl derivatives. Effects of acrolein, cinnamaldehyde, citral, crotonaldehyde, curcumin, ethacrynic acid, and trans-2-hexenal. Chem. Biol. Interact..

[107] Udenigwe C.C., Ata A., Samarasekera R. (2007). Glutathione *S*-Transferase Inhibiting Chemical Constituents of Caesalpinia bonduc. Chem. Pharm. Bull..

[108] Duvoix A., Morceau F., Delhalle S., Schmitz M., Schnekenburger M., Galteau M.-M., Dicato M., Diederich M. (2003). Induction of apoptosis by curcumin: Mediation by glutathione S-transferase P1-1 inhibition. Biochem. Pharmacol..

[109] Zaid M.A., Dalmizrak O., Teralı K., Ozer N. (2023). Mechanistic insights into the inhibition of human placental glutathione S-transferase P1-1 by abscisic and gibberellic acids: An integrated experimental and computational study. J. Mol. Recognit..

[110] Stärkel P., De Saeger C., Delire B., Magat J., Jordan B., Konda V.R., Tripp M.L., Borbath I. (2015). Tetrahydro Iso-Alpha Acids and Hexahydro Iso-Alpha Acids from Hops Inhibit Proliferation of Human Hepatocarcinoma Cell Lines and Reduce Diethylnitrosamine Induced Liver Tumor Formation in Rats. Nutr. Cancer.

[111] Wang B., Shen C., Li Y., Zhang T., Huang H., Ren J., Hu Z., Xu J., Xu B. (2019). Oridonin overcomes the gemcitabine resistant PANC-1/Gem cells by regulating GST pi and LRP/1 ERK/JNK signalling. Onco Targets Ther..

[112] van Haaften R.I.M., Haenen G.R.M.M., van Bladeren P.J., Bogaards J.J.P., Evelo C.T.A., Bast A. (2003). Inhibition of various glutathione S-transferase isoenzymes by RRR-alpha-tocopherol. Toxicol. Vitr..

[113] Wheeler J.B., Stourman N.V., Thier R., Dommermuth A., Vuilleumier S., Rose J.A., Armstrong R.N., Guengerich F.P. (2001). Conjugation of haloalkanes by bacterial and mammalian glutathione transferases: Mono- and dihalomethanes. Chem. Res. Toxicol..

[114] Olvera-Bello A.E., Estrada-Muñiz E., Elizondo G., Vega L. (2010). Susceptibility to the cytogenetic effects of dichloromethane is related to the glutathione S-transferase theta phenotype. Toxicol. Lett..

[115] Hu Y., Kabler S.L., Tennant A.H., Townsend A.J., Kligerman A.D. (2006). Induction of DNA–protein crosslinks by dichloromethane in a V79 cell line transfected with the murine glutathione-S-transferase theta 1 gene. Mutat. Res. Toxicol. Environ. Mutagen..

[116] Kayser M.F., Vuilleumier S. (2001). Dehalogenation of dichloromethane by dichloromethane dehalogenase/glutathione S-transferase leads to formation of DNA adducts. J. Bacteriol..

[117] Uppugunduri C.R.S., Muthukumaran J., Robin S., Santos-Silva T., Ansari M. (2022). In silico and In Vitro investigations on the protein–protein interactions of glutathione S-transferases with mitogen-activated protein kinase 8 and apoptosis signal-regulating kinase 1. J. Biomol. Struct. Dyn..

[118] Tew K.D. (2007). Redox in redux: Emergent roles for glutathione S-transferase P (GSTP) in regulation of cell signaling and S-glutathionylation. Biochem. Pharmacol..

[119] Tew K.D., Monks A., Barone L., Rosser D., Akerman G., Montali J.A., Wheatley J.B., Schmidt D.E. (1996). Glutathione-associated enzymes in the human cell lines of the National Cancer Institute Drug Screening Program. Mol. Pharmacol..

[120] Wang Z., Zhao Z., Cheng X., Liu S., Wei Q., Scott I.M. (2016). Conifer flavonoid compounds inhibit detoxification enzymes and synergize insecticides. Pestic. Biochem. Physiol..

[121] Mangoyi R., Hayeshi R., Ngadjui B., Ngandeu F., Bezabih M., Abegaz B., Razafimahefa S., Rasoanaivo P., Mukanganyama S. (2010). Glutathione transferase from Plasmodium falciparum--interaction with malagashanine and selected plant natural products. J. Enzyme Inhib. Med. Chem..

[122] Fakae B.B., Campbell A.M., Barrett J., Scott I.M., Teesdale-Spittle P.H., Liebau E., Brophy P.M. (2000). Inhibition of glutathione S-transferases (GSTs) from parasitic nematodes by extracts from traditional Nigerian medicinal plants. Phytother. Res..

[123] Horton J.K., Roy G., Piper J.T., Van Houten B., Awasthi Y.C., Mitra S., Alaoui-Jamali M.A., Boldogh I., Singhal S.S. (1999). Characterization of a chlorambucil-resistant human ovarian carcinoma cell line overexpressing glutathione S-transferase mu. Biochem. Pharmacol..

[124] Depeille P., Cuq P., Mary S., Passagne I., Evrard A., Cupissol D., Vian L. (2004). Glutathione S-transferase M1 and multidrug resistance protein 1 act in synergy to protect melanoma cells from vincristine effects. Mol. Pharmacol..

[125] Pandiyan A., Lari S., Vanka J., Kumar B.S., Ghosh S., Jee B., Jonnalagadda P.R. (2023). Genetic Polymorphism in Xenobiotic Metabolising Genes and Increased Oxidative Stress among Pesticides Exposed Agricultural Workers Diagnosed with Cancers. Asian Pac. J. Cancer Prev..

[126] Kumar K.V., Goturi A., Nagaraj M., Goud E.V.S.S. (2022). Null genotypes of Glutathione S-transferase M1 and T1 and risk of oral cancer: A meta-analysis. J. Oral Maxillofac. Pathol..

[127] Nesa A., Mostafijur Rahman M., Tahminur Rahman M., Kabir Y. (2023). Association of NAT2, GSTT1, and GSTM1 gene polymorphisms withprostate cancer risk in Bangladeshi population. Gene.

[128] Li S., Xue F., Zheng Y., Yang P., Lin S., Deng Y., Xu P., Zhou L., Hao Q., Zhai Z. (2019). GSTM1 and GSTT1 null genotype increase the risk of hepatocellular carcinoma: Evidence based on 46 studies. Cancer Cell Int..

[129] Heagerty A.H., Fitzgerald D., Smith A., Bowers B., Jones P., Fryer A.A., Zhao L., Alldersea J., Strange R.C. (1994). Glutathione S-transferase GSTM1 phenotypes and protection against cutaneous tumours. Lancet.

[130] Karimi E., Abbasnezhad S., Zeraattalab-Motlagh S., Amiri Khosroshahi R., Beh-Afarin S.R., Mohammadi H., Yaghmaie M. (2025). Diet, glutathione S-transferases M1 and T1 gene polymorphisms and cancer risk: A systematic review of observational studies. Br. J. Nutr..

[131] Rattan S.I.S. (2004). Mechanisms of hormesis through mild heat stress on human cells. Ann. N.Y. Acad. Sci..

[132] Matsukawa J., Matsuzawa A., Takeda K., Ichijo H. (2004). The ASK1-MAP kinase cascades in mammalian stress response. J. Biochem..

[133] Tibbles L.A., Woodgett J.R. (1999). The stress-activated protein kinase pathways. Cell. Mol. Life Sci..

[134] Cheng J.Z., Singhal S.S., Sharma A., Saini M., Yang Y., Awasthi S., Zimniak P., Awasthi Y.C. (2001). Transfection of mGSTA4 in HL-60 cells protects against 4-hydroxynonenal-induced apoptosis by inhibiting JNK-mediated signaling. Arch. Biochem. Biophys..

[135] Zhang Y., Talalay P., Cho C.G., Posner G.H. (1992). A major inducer of anticarcinogenic protective enzymes from broccoli: Isolation and elucidation of structure. Proc. Natl. Acad. Sci. USA.

[136] Kensler T.W., Wakabayashi N., Biswal S. (2007). Cell survival responses to environmental stresses via the Keap1-Nrf2-ARE pathway. Annu. Rev. Pharmacol. Toxicol..

[137] Su Z.-Y., Shu L., Khor T.O., Lee J.H., Fuentes F., Kong A.-N.T. (2012). A perspective on dietary phytochemicals and cancer chemoprevention: Oxidative stress, nrf2, and epigenomics. Nat. Prod. Cancer Prev. Ther..

[138] Zhang Y., Gordon G.B. (2004). A strategy for cancer prevention: Stimulation of the Nrf2-ARE signaling pathway. Mol. Cancer Ther..

[139] van Iersel M.L.P.S., Verhagen H., van Bladeren P.J. (1999). The role of biotransformation in dietary (anti) carcinogenesis. Mutat. Res. Toxicol. Environ. Mutagen..

[140] Surh Y.-J. (2003). Cancer chemoprevention with dietary phytochemicals. Nat. Rev. Cancer.

[141] Kundu J.K., Surh Y.-J. (2008). Cancer chemopreventive and therapeutic potential of resveratrol: Mechanistic perspectives. Cancer Lett..

[142] Cheng H., Zhang D., Wu J., Liu J., Zhou Y., Tan Y., Feng W., Peng C. (2023). Interactions between gut microbiota and polyphenols: A mechanistic and metabolomic review. Phytomedicine.

[143] Scalbert A., Williamson G. (2000). Dietary intake and bioavailability of polyphenols. J. Nutr..

[144] Manach C., Scalbert A., Morand C., Rémésy C., Jiménez L. (2004). Polyphenols: Food sources and bioavailability. Am. J. Clin. Nutr..

[145] Parl F.F. (2005). Glutathione S-transferase genotypes and cancer risk. Cancer Lett..

[146] Board P.G., Menon D. (2013). Glutathione transferases, regulators of cellular metabolism and physiology. Biochim. Biophys. Acta (BBA)-Gen. Subj..

